# Assessing the adequacy of essential nutrient intake in obese dogs undergoing energy restriction for weight loss: a cohort study

**DOI:** 10.1186/s12917-015-0570-y

**Published:** 2015-10-07

**Authors:** Alexander J. German, Shelley L. Holden, Samuel Serisier, Yann Queau, Vincent Biourge

**Affiliations:** Department of Obesity and Endocrinology (Holden and German), University of Liverpool, Leahurst Campus, Chester High Road, Neston, Wirral CH64 7TE UK; Royal Canin Research Center (Serisier, Queau, Biourge), 30470 Aimargues, France

**Keywords:** Canine, Caloric restriction, Nutritional adequacy

## Abstract

**Background:**

Canine obesity is usually treated with dietary energy restriction, but data are limited regarding nutritional adequacy. The aim of the current study was to compare intake of essential nutrients with National Research Council recommendations in obese dogs during weight management with a purpose-formulated diet.

**Methods:**

Twenty-seven dogs were included in this non-randomised retrospective observational cohort study. All were determined to be systemically well, and without significant abnormalities based upon physical examination and clinicopathological assessments. The dogs underwent a controlled weight loss protocol of at least 182 days’ duration using a high protein high fibre weight loss diet. Median, maximum, and minimum daily intakes of all essential nutrients were compared against NRC 2006 recommended allowances (RA) for adult dogs.

**Results:**

Median weight loss was 28 % (16–40 %), mean daily energy intake was 61 kcal/kg^0.75^ (44–74 kcal/kg^0.75^), and no clinical signs of nutrient deficiency were observed in any dog. Based upon the average nutrient content of the diet, daily intake of the majority of essential nutrients was greater than their NRC 2006 recommended allowance (RA per kg body weight^0.75^), except for selenium, choline, methionine/cysteine, tryptophan, magnesium, and potassium. However, apart from choline (2/27 dogs) and methionine/cysteine (2/27 dogs), all essential nutrients remained above NRC minimum requirements (MR) throughout the trial.

**Conclusions:**

When fed the diet used in the current study, daily intakes of most essential nutrients meet both their NRC 2006 RA and MR in obese dogs during weight loss. In light of absence of clinical signs of nutrient deficiency, it is unclear what significance intakes less that NRC cut-offs for some nutrients have (especially selenium and choline), and further studies are recommended.

**Electronic supplementary material:**

The online version of this article (doi:10.1186/s12917-015-0570-y) contains supplementary material, which is available to authorized users.

## Background

Obesity is a common problem in dogs [1], and is associated with numerous diseases [1–3], metabolic derangements [4], alterations in renal function [5], respiratory dysfunction [6, 7], decreased longevity [8], and decreased quality of life [9]. Management involves controlled weight loss by caloric restriction using a purpose-formulated weight loss diet [10–14]. Such diets are designed to ensure delivery of all essential nutrients appropriate to daily requirements, despite sufficient energy restriction to promote weight loss. Such endeavours can be challenging in light of the fact that the exact level of energy restriction required can vary considerably, with some dogs requiring marked caloric restriction [11, 12].

To ensure a diet is complete and balanced when fed at maintenance requirements, it is recommended that daily intake of all essential nutrients meet internationally accepted recommendations, such as those reported by National Research Council (NRC) [15], the Association of American Feed Control Officials (AAFCO) [16], and the European Pet Food Industry Federation (FEDIAF) [17]. It is currently assumed that dogs undergoing weight management continue to have similar essential nutrient requirements to those required for maintenance, although there is limited evidence to support this supposition. A recent study demonstrated that plasma concentrations of most essential nutrients do not change significantly during controlled weight loss using a purpose-formulated diet [18]. Furthermore, no clinically-evident nutrient deficiencies have previously been reported from a number of studies that have assessed the weight management process [10–14]. However, a recent theoretical study suggested the theoretical possibility that intake of some nutrients could be insufficient during the weight loss process, depending upon the diet used and the degree of energy restriction [19]. The main limitation of this study was the fact that all estimates of nutrient intake were theoretical, rather than being determined from the food intake of dogs actually undergoing weight management. As a result, the aim of the current study was to compare intake of essential nutrients with NRC recommendations in obese dogs during weight management with a purpose-formulated diet.

## Methods

### Study design

This was a non-randomised retrospective observational cohort study to assess intake of selected nutrients in a cohort of dogs with naturally occurring obesity, and has been reported according to the Strengthening and Reporting of Observational Studies in Epidemiology (STROBE) statement guidelines (Additional file 1) [20].

### Animals

Participating dogs were recruited from referrals to the Royal Canin Weight Management Clinic, University of Liverpool UK, for management of obesity. The dogs were recruited between September 2006 and August 2010, and had completed weight loss by February 2011. To be eligible, all dogs had to be obese (based upon body fat measurement by dual-energy X-ray absorptiometry; DEXA) [11, 12], to have completed a weight loss regime (i.e. reaching target weight) of at least 182 days’ duration (i.e. ~6 months duration), to have been fed the same purpose-formulated weight loss diet (Table 1; Royal Canin Satiety Canine Dry Diet, Royal Canin, UK), to be euthyroid (based upon measurement of serum free thyroxine concentration by equilibrium dialysis), and to have had no significant systemic disease (e.g. endocrine disease, hepatic disease, renal disease, and gastrointestinal disease) during the study. The study protocol adhered to the University of Liverpool Animal Ethics Guidelines, and was approved by both the University of Liverpool Research Ethics Committee and the Royal Canin Ethical Review Committee. Owners of all participating animals gave informed consent in writing.

**Table 1:** Average nutrient content of the diet used for weight loss in the study dogs

*Nutrient* ^*a*^	Weight loss diet	
*Kcal/kg Metabolisable Energy* ^*b*^	2900	---
	Per 100 g as fed	Per 1000 kcal
*Crude protein (g)*	30	104.0
*Arginine (g)*	1.6	5.4
*Histidine (g)*	0.6	2.0
*Isoleucine (g)*	1.1	3.8
*Met and Cys (g)*	1.0	3.6
*Leucine (g)*	2.2	7.7
*Lysine (g)*	1.2	4.1
*Phe and Tyr (g)*	2.8	9.6
*Threonine (g)*	1.0	3.3
*Tryptophan (g)*	0.3	0.9
*Taurine (g)* ^*c*^	0.2	0.7
*Valine (g)*	1.3	4.4
*Total fat (g)*	9.6	33.0
*Linoleic acid (g)*	2.1	7.3
*Calcium (g)*	0.9	3.1
*Phosphorus (g)*	0.7	2.4
*Magnesium (g)*	0.05	0.2
*Sodium (g)*	0.3	1.0
*Potassium (g)*	0.8	2.8
*Chloride (g)*	0.9	3.0
*Iron (mg)*	16.5	57.0
*Copper (mg)*	2.0	6.9
*Zinc (mg)*	20.0	69.0
*Manganese (mg)*	7.0	24.0
*Selenium (mg)*	0.02	0.1
*Iodine (mg)*	0.3	1.0
*Vitamin A (IU)*	2027	6990
*Vitamin D3 (IU)*	70	24
*Vitamin E (IU)*	80	276
*Thiamine (mg)*	2.6	9.0
*Riboflavin (mg)*	5.2	18.0
*Pyridoxine (mg)*	2.3	8.0
*Niacin (mg)*	16	56
*Pantothenic acid (mg)*	4.6	16.0
*Cobalamin (mg)*	0.02	0.06
*Folic acid (mg)*	0.4	1.5
*Choline (mg)*	249	860

^a^The values reported are the average nutrient content as analysed

^b^Metabolisable energy content, as measured in animal trials according to the AAFCO protocol [16]

^c^Although taurine is not an essential amino acid, it is supplemented within the diet

### Weight loss regimen

Full details of the weight loss regimen have been previously described [11, 12]. Briefly, dogs were determined to be systemically well, and without significant abnormalities based upon physical examination and clinicopathological assessments (as detailed above). A controlled weight loss protocol was then initiated, using a high protein high fibre weight loss diet (Table 1). The initial food allocation for weight loss was determined by first estimating maintenance energy requirement (MER = 440 kJ [105Kcal] × body weight [kg]^0.75^/day [21]) using the estimated target weight. The exact level of restriction for each dog was then individualised based upon gender, and was typically between 50–60 % of MER at target weight [11]. Owners also implemented lifestyle and activity alterations to assist in weight loss. Dogs were reweighed every 7–21 days and changes made to the food allocation if necessary.

Throughout weight loss, patients were weighed on electronic scales (Soehnle Professional, Backnang, Germany), which were regularly calibrated using test weights (Blake and Boughton Ltd, Thetford, UK). At the end of the weight management period, a detailed re-evaluation was conducted which included measurement of body weight, blood sampling, urinalysis, blood pressure measurement, and assessment of body composition assessed by fan-beam DEXA (Lunar Prodigy Advance; GE Lunar, Madison, Wisc, USA) [11, 22].

### Estimation of nutrient intake

Average, maximum and minimum intakes of each nutrient were calculated for the period of weight management for each dog. Maximum and minimum daily intakes were defined as the greatest and least daily intake that the dog received during the period of controlled weight loss, whilst the average daily intake was defined as the mean daily intake for the whole weight loss period. The number (and percentage) of dogs with daily intake of essential nutrients less than NRC 2006 recommendations (per kg of ideal body weight^0.75^) was then determined, whereby minimum requirement (MR) was defined as ‘the minimal concentration of a maximally bioavailable nutrient that will support a defined physiological state’, recommended allowance (RA) was defined as ‘the concentration of nutrient demonstrated to support a defined physiological state’, and adequate intake (AI) was defined as ‘the concentration of nutrient demonstrated to support a defined physiological state when no MR has been demonstrated’ [15].

### Data handling and analysis

All study data are available in the supplemental material (see Additional file 2). Absolute data are expressed as median (range). Datasets were complete for most variables except for four serum biochemistry variables where small numbers of results were missing: alkaline phosphatase (pre-weight-loss: 1 result), glucose (pre-weight-loss: 2 results; post-weight-loss: 3 results), phosphate (pre-weight-loss: 1 result), potassium (post-weight-loss: 2 results), and free thyroxine (post-weight-loss: 2 results). There were no other missing data for any variable. Given the small numbers of missing data points, no correction was made in statistical analysis. For each dog, the daily intake of all essential nutrients was calculated, based upon their food allocation and the average nutrient content of the diet (Table 1). Statistical analyses were performed with computer software (Stats Direct version 2.6.8, Stats Direct Ltd), with the level of significance set at *P* < 0.05 for two-sided analyses. Given that there were no known previous studies examining adequacy of essential nutrient intake in obese dogs undergoing weight loss, it was not possible to perform a meaningful power calculation. Instead, as many dogs as possible were enrolled that the met the eligibility criteria. The Shapiro-Wilk test was used to assess all data sets and, because many were not normally distributed, non-parametric tests were used in preliminary analyses. These included the signed ranks test, the Mann–Whitney *U* test, Kendall’s rank correlation, and Fisher’s exact test. In addition, simple and multiple regression analysis was used to determine associations between variables, and the Shapiro-Wilk test was also used to confirm that the distribution of the residuals of each regression model followed a normal distribution. Variables assessed included baseline variables (e.g. age, sex, neuter status, breed, and body fat percentage) and weight loss parameters (e.g. percentage weight loss, rate of weight loss, change in lean tissue, and energy intake during weight loss).

For linear regression of changes in lean tissue mass and circulating albumin concentration, factors tested included signalment (e.g. age at enrolment, sex, breed group [retriever vs. not retriever]), baseline parameters (e.g. percentage body fat pre-weight-loss, starting body weight, lean tissue mass pre-weight loss [in kg]), weight loss outcomes (duration of weight loss, percentage weight loss, energy intake during weight loss, and percentage change in body fat mass), and nutrient status. For the latter, dogs were classified according to a binary variable, whereby 0 = meets its NRC 2006 RA, and 1 = does not meet its NRC 2006 RA. Both the average daily intake and the minimum daily intake during the weight loss period were assessed separately. Percentage change in fat mass was calculated using the following formula: (fat mass [kg]_POST_ – fat mass [kg]_PRE_) ÷ fat mass [kg]_PRE_ × 100); similarly, percentage change in lean mass was calculated as: (lean mass [kg]_POST_ – lean mass [kg]_PRE_) ÷ lean mass [kg]_PRE_ × 100). Initially, simple linear regression was used. A multiple linear regression model was then constructed, which initially included any variables identified as *P* < 0.2 on univariable analysis. Collinearity amongst variables was assessed such that unnecessary collinear predictors were removed. The model was then refined by backwards-stepwise elimination of the least significant variable at each round, with variables being retained in the final model if they were significant (*P* < 0.05).

## Results

### Dogs and outcome of weight loss

During the study timeframe, a total of 149 dogs were enrolled in a weight loss regime and potentially eligible for study recruitment. Of these, 82 dogs reached their target weight, and the period of weight management was at least 182 days in 52 dogs. Of these 52 dogs, 40 had been fed a single type of dry food (Table 1). After excluding dogs that were not euthyroid, were not on concurrent medication, or had incomplete clinical data (laboratory and DEXA scan results), a final group of 27 dogs were eligible for inclusion. These dogs represented a range of ages, breeds and sexes (Table 2), and the median period of weight loss was 293 days (range 182–674 days). All dogs remained well throughout, and no diet-related abnormalities were noted on physical examination, before, during, and after the period of weight loss. Median weight loss was 28.3 % (16.0-40.1 %) starting body weight (SBW), with a median rate of 0.6 % (0.2-1.4 %) SBW/week, and the bulk of the tissue lost was fat with a lesser amount of lean tissue (Table 2). The mean daily energy intake during weight loss was 61 Kcal/kg^0.75^ (44–74 Kcal/kg^0.75^), maximum daily energy intake was 63 Kcal/kg^0.75^ (45–77 Kcal/kg^0.75^), and minimum daily energy intake was 60 Kcal/kg^0.75^ (43–71 Kcal/kg^0.75^).

**Table 2:** Summary of weight loss in the study dogs

Criterion	Result
Age (months)	70 (24 to 228)
Sex	1 M, 16 NM, 1 F, 9 NF
Breed	Alaskan Malamute, Border Collie, CKCS (4), Cocker Spaniel, Corgi, Dachshund, Doberman (2), Golden Retriever, Irish Setter, Labrador (6), Mixed Breed (3), Pug (2), Samoyed, Yorkshire Terrier (2)
Body weight PRE (kg)	32.9 (6.7 to 66.8)
Body weight POST (kg)	23.2 (5.0 to 48.0)
Body fat mass PRE (%)	47 (31 to 55)
Body fat mass POST (%)	30 (19 to 45)
Duration (days)	293 (182 to 674)
Rate of weight loss (%/week)^a^	0.6 (0.2 to 1.4)
Body weight change (%)^b^	−28.3 (−16.0 to −40.1)
Change in fat mass (%)^b^	−52 (−67 to −16)
Change in lean mass (%)^b^	−10 (−21 to +5)
EI during weight loss^c^	256 (184 to 308) [61 (44 to 74)]

All data are expressed as median (range). M: male; NM: neutered male; F: female; NF: neutered female; CKCS: Cavalier King Charles Spaniel

^a^Rate of weight loss expressed as percentage of starting body weight lost per week

^b^Refers to the percentage change in starting mass calculated as follows: ([start mass – end mass] ÷ start mass) ×100%

^c^EI: energy intake expressed as metabolisable energy (in kJ [Kcal]) per kg of metabolic body weight (BW^0.75^) per day

### Laboratory assessments

All dogs remained euthyroid throughout the trial, and serum free thyroxine concentration did not change significantly from beginning to end (Table 3; *P* = 0.562). Routine haematological and serum biochemical analyses were also performed (Table 3); occasional results were marginally outside the reference range, as reported in similar studies [4, 5, 18]. Also as previously reported [4, 5, 18], white blood cell counts, and concentrations of albumin, calcium, cholesterol, creatinine, and globulins all decreased during weight loss, whilst urea concentration increased (Table 3). However, for the most part, changes were minor, and the majority remained within the reference range.

**Table 3:** Haematological and clinical biochemical results before and after weight loss

*Parameter*	Before weight loss	After weight loss	Reference range	*P* value
*Red blood cells ×10* ^*12*^ */L*	6.9 (5.3-8.1), 0, 2	6.9 (5.3-8.0), 0, 1	5.5-8.2	0.652
*Haemoglobin g/L*	16.0 (12.4-18.8), 0, 2	16.0 (11.3-17.9), 0, 3	12.6-19.4	0.690
*Haematocrit L/L*	0.48 (0.36-0.55), 0, 0	0.47 (0.35-0.54), 0, 0	0.35-0.55	0.880
*Platelets* *×10* ^*9*^ */L*	276 (13–385), 0, 2	266 (140–444), 0, 0	80-560	0.473
*White blood cells ×10* ^*9*^ */L*	10.4 (5.1-18.7), 2, 1	8.2 (4.6-14.0), 0, 5	6.7-18.3	<0.001
*Sodium mmol/L*	147 (142–153), 0, 0	146 (140–151), 0, 0	140-153	0.155
*Potassium mmol/L*	4.6 (3.8-6.0), 1, 0	4.2 (3.7-6.0), 1, 1	3.8-5.3	0.199
*Calcium mmol/L*	2.8 (2.3-3.1), 3, 0	2.6 (1.8-2.9), 0, 2	2.20-2.90	<0.001
*Phosphate mmol/L*	1.0 (0.5-2.2), 1, 3	1.0 (0.5-1.5), 0, 5	0.8-2.0	0.058
*Alanine aminotransferase U/L*	44 (18–293), 13, 0	49 (18–238), 13, 0	7-50	0.893
*Alkaline phosphatase U/L*	74 (23–392), 11, 0	54 (26–295), 8, 0	0-100	0.065
*Albumin g/L*	32 (28–36), 1, 0	29 (25–36), 2, 0	25-35	<0.001
*Cholesterol mmol/L*	6.1 (4.1-7.9), 5, 0	5.4 (3.0-7.3), 1, 2	3.5-7.0	0.040
*Creatinine μmol/L*	84 (44–123), 2, 0	72 (46–114), 1, 0	20-110	0.033
*Globulins g/L*	31 (22–44), 2, 0	27 (23–42), 1, 0	22-40	0.012
*Glucose mmol/L*	5.1 (3.3-5.9), 5, 1	5.2 (3.0-7.4), 5, 1	3.5-5.5	0.210
*Urea mmol/L*	4.6 (1.8-7.6), 4, 5	5.2 (2.4-8.9), 1, 1	3.5-7.0	0.047
*Free thyroxine pmol/L*	21.0 (7.0-42.9), 2, 0	20.0 (8.4-34.0), 0, 0	6.6-40.0	0.562

Data are expressed as median (range), number of results above reference range, and number below reference range

*P* values quoted for Wilcoxon signed ranks test comparing pre- and post-weight-loss measurements. In the dogs, before and after weight loss, with low platelet counts, platelet clumps were identified on a blood smear suggesting that actual numbers were normal

### Comparison with National Research Council 2006 recommendations

Average, maximum and minimum daily intakes of essential nutrients for each dog (expressed per kg BW^0.75^) were calculated for the whole weight management period (Table 4), and compared with NRC 2006 recommendations (Table 5). The intake of the majority of essential nutrients was greater than all NRC 2006 cut-offs, with the exception of selenium, choline, methionine and cysteine, tryptophan, total fat, magnesium, and potassium. For selenium, the minimum daily intake was less than both RA and AI in all dogs. For choline, the minimum daily intake was less than RA in 24/27 dogs (89 %), but less than AI in only 2 dogs (7 %). For methionine and cysteine, the minimum daily intake was less than RA in 12 dogs (44 %), but minimum intake was never less than MR in only 2 dogs (7 %). Finally, minimum daily intake was less than RA for tryptophan (2 dogs, 7 %), magnesium (27 dogs, 100 %), and potassium (2 dogs, 7 %), but was never less than MR in any dog.

**Table 4:** Daily intake of essential nutrients in 27 obese dogs during weight loss

*Nutrient*	Daily intake		
	Maximum^a^	Minimum^b^	Average^c^
*Crude protein (g)*	6.55 (4.70-8.04)	6.23 (4.46-7.38)	6.35 (4.58-7.64)
*Arginine (g)*	0.342 (0.245-0.419)	0.324 (0.232-0385)	0.331 (0.239-0.398)
*Histidine (g)*	0.129 (0.092-0.158)	0.122 (0.087-0.145)	0.125 (0.090-0.150)
*Isoleucine (g)*	0.237 (0.170-0.291)	0.225 (0.161-0.267)	0.230 (0.166-0.276)
*Methionine & cysteine (g)*	0.22 (0.16-0.28)	0.21 (0.15-0.25)	0.22 (0.16-0.26)
*Leucine (g)*	0.49 (0.35-0.60)	0.46 (0.33-0.55)	0.47 (0.34-0.57)
*Lysine (g)*	0.259 (0.186-0.318)	0.246 (0.176-0.292)	0.251 (0.181-0.302)
*Phenylalanine & tyrosine (g)*	0.61 (0.44-0.74)	0.58 (0.41-0.68)	0.59 (0.42-0.71)
*Threonine (g)*	0.21 (0.15-0.26)	0.20 (0.14-0.23)	0.20 (0.15-0.24)
*Tryptophan (g)*	0.059 (0.042-0.072)	0.056 (0.040-0.066)	0.057 (0.041-0.068)
*Valine (g)*	0.27 (0.20-0.34)	0.26 (0.19-0.31)	0.27 (0.19-0.32)
*Linoleic acid (g)*	0.46 (0.33-0.56)	0.43 (0.31-0.51)	0.44 (0.32-0.53)
*Calcium (g)*	0.195 (0.140-0.240)	0.186 (0.133-0.220)	0.189 (0.136-0.228)
*Phosphorus (g)*	0.15 (0.11-0.19)	0.14 (0.10-0.17)	0.15 (0.11-0.18)
*Magnesium (mg)*	10.7 (7.7-13.1)	10.2 (7.3-12.1)	10.4 (7.5-12.5)
*Sodium (mg)*	63.0 (45.2-77.3)	59.9 (42.8-71.0)	61.0 (44.0-73.5)
*Potassium (g)*	0.18 (0.13-0.22)	0.17 (0.12-0.20)	0.17 (0.12-0.21)
*Chloride (mg)*	189 (136–232)	180 (129–213)	183 (132–221)
*Iron (mg)*	3.6 (2.6-4.4)	3.4 (2.4-4.0)	3.5 (2.5-4.2)
*Copper (mg)*	0.4 (0.3-0.5)	0.4 (0.3-0.5)	0.4 (0.3-0.5)
*Zinc (mg)*	4.3 (3.1-5.3)	4.1 (3.0-4.9)	4.2 (3.0-5.1)
*Manganese (mg)*	1.5 (1.1-1.9)	1.4 (1.0-1.7)	1.5 (1.1-1.8)
*Selenium (μg)*	5.1 (3.7-6.1)	5.2 (3.8-6.4)	5.0 (3.6-5.9)
*Iodine (mg)*	63.0 (45.2-77.3)	59.9 (42.8-71.0)	61.0 (44.0-73.5)
*Vitamin A (IU)*	440 (316–541)	418 (299–496)	427 (308–514)
*Vitamin D3 (IU)*	15.25 (10.94-18.71)	14.49 (10.37-17.18)	14.77 (10.65-17.79)
*Vitamin E (IU)*	17.4 (12.5-21.3)	16.5 (11.8-19.6)	16.8 (12.2-20.3)
*Thiamine (mg)*	0.567 (0.407-0.696)	0.539 (0.386-0.639)	0.549 (0.396-0.662)
*Riboflavin (mg)*	1.134 (0.814-1.392)	1.078 (0.771-1.278)	1.099 (0.792-1.323)
*Pyridoxine (mg)*	0.504 (0.362-0.619)	0.479 (0.343-0.568)	0.488 (0.352-0.588)
*Niacin (mg)*	3.53 (2.53-4.33)	3.35 (2.40-3.97)	3.42 (2.47-4.12)
*Pantothenic acid (mg)*	1.01 (0.72-1.24)	0.96 (0.69-1.14)	0.98 (0.70-1.18)
*Cobalamin (μg)*	3.47 (2.49-4.25)	3.29 (2.36-3.90)	3.36 (2.42-4.04)
*Folic acid (μg)*	94.5 (67.8-116.0)	89.8 (64.3-106.5)	91.6 (66.0-110.3)
*Choline (mg)*	54.2 (38.9-66.5)	51.5 (36.8-61.0)	52.5 (37.9-63.2)

The daily intake of all essential nutrients was calculated from their food allocation and the average nutrient content of the diet (Table 1), and expressed per kg BW^0.75^. Results are quoted as median (range)

^a^Maximum daily intake defined as the greatest daily intake that the dog received during the period of controlled weight loss

^b^Minimum daily intakes defined as the least daily intake that the dog received during the period of controlled weight loss

^c^Mean daily intake was defined as the mean daily intake for the whole period

**Table 5:** Obese dogs not meeting NRC requirements during weight loss

*Nutrient*	Recommended allowance^a^			Adequate intake^b^			Minimum requirement^c^		
	*NRC* ^*d*^	No. less^e^	%^f^	*NRC*	No. less^e^	%^f^	*NRC*	No. less^e^	%^f^
*Crude protein (g)*	*3.28*	0, 0, 0	0, 0, 0	*---*	---	---	*2.62*	0, 0, 0	0, 0, 0
*Arginine (g)*	*0.110*	0, 0, 0	0, 0, 0	*---*	---	---	*0.092*	0, 0, 0	0, 0, 0
*Histidine (g)*	*0.062*	0, 0, 0	0, 0, 0	*---*	---	---	*0.048*	0, 0, 0	0, 0, 0
*Isoleucine (g)*	*0.120*	0, 0, 0	0, 0, 0	*---*	---	---	*0.098*	0, 0, 0	0, 0, 0
*Methionine & cysteine (g)*	*0.21*	7, 12, 9	26, 44, 33	*---*	---	---	*0.17*	1, 2, 1	4, 7, 4
*Leucine (g)*	*0.22*	0, 0, 0	0, 0, 0	*---*	---	---	*0.18*	0, 0, 0	0, 0, 0
*Lysine (g)*	*0.110*	0, 0, 0	0, 0, 0	*---*	---	---	*0.092*	0, 0, 0	0, 0, 0
*Phenylalanine & tyrosine (g)*	*0.24*	0, 0, 0	0, 0, 0	*---*	---	---	*0.19*	0, 0, 0	0, 0, 0
*Threonine (g)*	*0.14*	0, 0, 0	0, 0, 0	*---*	---	---	*0.11*	0, 0, 0	0, 0, 0
*Tryptophan (g)*	*0.046*	1, 2, 1	4, 7, 4	*---*	---	---	*0.036*	0, 0, 0	0, 0, 0
*Valine (g)*	*0.16*	0, 0, 0	0, 0, 0	*---*	---	---	*0.13*	0, 0, 0	0, 0, 0
*Linoleic acid (g)*	*0.36*	0, 0, 0	0, 0, 0	*0.30*	0, 0, 0	0, 0, 0	*---*	---	---
*Calcium (g)*	*0.130*	0, 0, 0	0, 0, 0	*---*	---	---	*0.059*	0, 0, 0	0, 0, 0
*Phosphorus (g)*	*0.10*	0, 0, 0	0, 0, 0	*0.10*	0, 0, 0	0, 0, 0	*---*	---	---
*Magnesium (mg)*	*19.70*	27, 27, 27	100,100,100	*---*	---	---	*5.91*	0, 0, 0	0, 0, 0
*Sodium (mg)*	*26.2*	0, 0, 0	0, 0, 0	*---*	---	---	*9.85*	0, 0, 0	0, 0, 0
*Potassium (g)*	*0.14*	1, 2, 1	4, 7, 4	*0.14*	1, 2, 1	4, 7, 4	*---*	---	---
*Chloride (mg)*	*40*	0, 0, 0	0, 0, 0	*40*	0, 0, 0	0, 0, 0	*---*	---	---
*Iron (mg)*	*1.0*	0, 0, 0	0, 0, 0	*1.0*	0, 0, 0	0, 0, 0	*---*	---	---
*Copper (mg)*	*0.2*	0, 0, 0	0, 0, 0	*0.2*	0, 0, 0	0, 0, 0	*---*	---	---
*Zinc (mg)*	*2.0*	0, 0, 0	0, 0, 0	*2.0*	0, 0, 0	0, 0, 0	*---*	---	---
*Manganese (mg)*	*0.16*	0, 0, 0	0, 0, 0	*0.16*	0, 0, 0	0, 0, 0	*---*	---	---
*Selenium (μg)*	*11.8*	27, 27, 27	100,100,100	*11.8*	27, 27, 27	100,100,100	*---*	---	---
*Iodine (mg)*	*29.6*	0, 0, 0	0, 0, 0	*---*	---	---	*23.6*	0, 0, 0	0, 0, 0
*Vitamin A (IU)*	*50*	0, 0, 0	0, 0, 0	*40*	0, 0, 0	0, 0, 0	*---*	---	---
*Vitamin D3 (IU)*	*0.45*	0, 0, 0	0, 0, 0	*0.36*	0, 0, 0	0, 0, 0	*---*	---	---
*Vitamin E (IU)*	*1.0*	0, 0, 0	0, 0, 0	*0.8*	0, 0, 0	0, 0, 0	*---*	---	---
*Thiamine (mg)*	*0.074*	0, 0, 0	0, 0, 0	*0.059*	0, 0, 0	0, 0, 0	*---*	---	---
*Riboflavin (mg)*	*0.171*	0, 0, 0	0, 0, 0	*---*	---	---	*0.138*	0, 0, 0	0, 0, 0
*Pyridoxine (mg)*	*0.049*	0, 0, 0	0, 0, 0	*---*	---	---	*0.040*	0, 0, 0	0, 0, 0
*Niacin (mg)*	*0.49*	0, 0, 0	0, 0, 0	*0.39*	0, 0, 0	0, 0, 0	*---*	---	---
*Pantothenic acid (mg)*	*0.49*	0, 0, 0	0, 0, 0	*0.40*	0, 0, 0	0, 0, 0	*---*	---	---
*Cobalamin (μg)*	*1.15*	0, 0, 0	0, 0, 0	*0.92*	0, 0, 0	0, 0, 0	*---*	---	---
*Folic acid (μg)*	*8.9*	0, 0, 0	0, 0, 0	*7.1*	0, 0, 0	0, 0, 0	*---*	---	---
*Choline (mg)*	*56.0*	17, 24, 20	63, 89, 74	*45.0*	1, 2, 1	4, 7, 4	*---*	---	---

^a^Recommended allowance: the concentration of nutrient demonstrated to support a defined physiological state [15]

^b^Adequate intake: the concentration of nutrient demonstrated to support a defined physiological state when no minimal requirement has been demonstrated [15]

^c^Minimum requirement: the minimal concentration of a maximally bioavailable nutrient that will support a defined physiological state [15]

^d^All requirements are expressed as the unit stated per kg BW^0.75^

^e^The number of dogs with nutrient intakes less than NRC cut-off based upon maximum, minimum, and mean daily intakes, respectively

^f^The percentage of dogs with nutrient intakes less than NRC cut-off based upon maximum, minimum, and mean daily intakes, respectively. Please see the footnote of Table 4 for definitions of maximum, minimum and mean intakes

### Association between nutrients with borderline intakes and changes in either lean tissue and serum albumin concentration

Given that, in some dogs, intake of certain nutrients was borderline, the possibility that these might be associated with changes in lean tissue mass and circulating albumin concentration was explored using linear regression. Selenium was not assessed separately because all dogs had intakes less than RA and, therefore, this factor did not discriminate dogs. On simple linear regression analysis (Table 6), one factor (percentage weight loss) was significantly associated with change in lean tissue during weight loss (*R* = −0.55, *R*^*2*^ = 0.30, *P* = 0.003), whilst a number of other factors were also eligible for inclusion in the preliminary multiple linear regression model (*P* < 0.2) including: age (*R* = −0.27, *R*^*2*^ = 0.07, *P* = 0.170), rate of weight loss (*R* = −0.33, *R*^*2*^ = 0.11, *P* = 0.092), energy intake (*R* = 0.28, *R*^*2*^ = 0.08, *P* = 0.152), average daily intake of methionine and cysteine (*R* = −0.32, *R*^*2*^ = 0.10, *P* = 0.107), and minimum daily intake of choline mean (*R* = −0.31, *R*^*2*^ = 0.10, *P* = 0.111). After refinement of the initial model by backwards stepwise elimination, the best-fit model was one that included a single variable, percentage weight loss (more lean tissue loss when greater percentage weight loss; *R* = −0.55, *R*^*2*^ = 0.31, *P* = 0.003; Table 6). Thus, essential nutrient intake was not associated with changes in lean tissue during weight loss.

**Table 6:** Simple and multiple linear regression analysis to determine factors associated with change in lean mass

Variable	R	R^2^	Probability
Simple regression			
Age^a^	−0.27	0.07	0.170
Sex	0.09	0.01	0.658
Neuter status	−0.14	0.02	0.471
Duration of weight loss	0.057	0.00	0.781
Percentage weight loss	−0.55	0.30	0.003
Mean EI during weight loss^b^	0.28	0.08	0.152
Mean rate of weight loss^c^	−0.33	0.11	0.092
Starting percentage body fat	−0.28	0.08	0.152
Change in fat mass^d^	0.11	0.01	0.605
Mean daily intake < RDA^e^			
Methionine & Cysteine	−0.32	0.10	0.107
Tryptophan	−0.21	0.05	0.282
Choline	−0.13	0.02	0.514
Minimum daily intake < RDA^f^			
Methionine & Cysteine	−0.21	0.05	0.282
Tryptophan	0.05	0.00	0.790
Choline	−0.31	0.10	0.111
Final multiple regression model			
Percentage weight loss	−0.55	0.30	0.003

All data are expressed as median (range). EI: energy intake; RDA: recommended daily allowance

^a^Age at the start of the weight loss programme

^b^Mean energy intake expressed as metabolisable energy (in kJ [Kcal]) per kg of metabolic body weight (BW^0.75^) per day

^c^Mean rate of weight loss expressed as percentage of starting body weight per week

^d^Percentage change in fat mass between the start and end of the weight loss period

^e^Dummy variable created whereby 1 = the mean daily intake of each nutrient less than the NRC 2006 RA, and 0 when it was greater [15]

^f^Dummy variable created whereby 1 was assigned when the minimum daily intake of the nutrient less than the NRC 2006 RA, and 0 was assigned when it was greater [15]

Simple linear regression analysis revealed that only the duration of weight loss was significantly associated with changes in serum albumin concentration (*R* = −0.39, *R*^*2*^ = 0.15, *P* = 0.046). Two other factors also qualified for inclusion in the preliminary multiple regression model: body fat prior to weight loss (*R* = −0.28, *R*^*2*^ = 0.08, *P* = 0.150), and the minimum daily intake of choline during weight loss (*R* = 0.26, *R*^*2*^ = 0.07, *P* = 0.184). Once again, after refinement by backwards stepwise elimination, the best-fit model was one that included the single variable, duration of weight loss (the longer the weight loss period, the greater the decrease in albumin; *R* = −0.39, *R*^*2*^ = 0.15, *P* = 0.046; Table 7). Thus, essential nutrient intake was not associated with changes in albumin concentration during weight loss.

**Table 7:** Simple and multiple linear regression analysis to determine factors associated with change in serum albumin concentration

Variable	R	R^2^	Probability
Simple regression			
Age^a^	0.22	0.05	0.274
Sex	0.18	0.03	0.357
Neuter status	0.01	0.00	0.956
Duration of weight loss	−0.39	0.15	0.046
Percentage weight loss	0.01	0.00	0.941
Mean EI during weight loss^b^	0.04	0.00	0.856
Mean rate of weight loss^c^	0.19	0.04	0.344
Starting percentage body fat	−0.28	0.08	0.150
Change in fat mass^d^	−0.02	0.00	0.921
Change in lean mass^d^	−0.07	0.00	0.718
Mean daily intake < RDA^e^			
Methionine & Cysteine	−0.12	0.01	0.566
Tryptophan	−0.03	0.00	0.895
Choline	−0.07	0.00	0.714
Minimum daily intake < RDA^f^			
Methionine & Cysteine	−0.14	0.02	0.482
Tryptophan	−0.03	0.00	0.894
Choline	0.26	0.07	0.184
Final multiple regression model			
Duration of weight loss	−0.39	0.15	0.046

All data are expressed as median (range). EI: energy intake; RDA: recommended daily allowance

^a^Age at the start of the weight loss programme

^b^Mean energy intake expressed as metabolisable energy (in kJ [Kcal]) per kg of metabolic body weight (BW^0.75^) per day

^c^Mean rate of weight loss expressed as percentage of starting body weight per week

^d^Percentage change in fat or lean mass between the start and end of the weight loss period; positive and negative values represent gains and losses in lean mass, respectively

^e^Dummy variable created whereby 1 = the mean daily intake of each nutrient less than the NRC 2006 RA, and 0 when it was greater [15]

^f^Dummy variable created whereby 1 was assigned when the minimum daily intake of the nutrient less than the NRC 2006 RA, and 0 was assigned when it was greater [15]

## Discussion

In the current study, we have assessed the intake of essential nutrients for obese dogs during a period of controlled weight loss using a purpose-formulated diet. The intake of most essential nutrients exceeded NRC 2006 RA cut-offs. Although intake was less than RA or MR for some nutrients, all dogs remained healthy, showing no clinical signs of nutrient deficiency. It is important to note that RAs include a safety margin to take into account predicted nutrient bioavailability, and the actual bioavailability of individual nutrients might be higher than assumed in NRC 2006. The significance of dogs having intakes less than MR for some nutrients during weight loss is not known. Firstly, it is possible that requirements for essential nutrients might actually change when dogs are subjected to energy restriction, although the exact effect may well differ amongst nutrients. For example, requirements of many amino acids and B vitamins are directly related to energy metabolism [15, 23]. Given that MER declines during weight loss [13], the requirement of some essential nutrients might also decline. In contrast, requirements for other essential nutrients (i.e. minerals) are more directly related to bodyweight, or another exponent, rather than to energy metabolism [15]. For these essential nutrients, requirements might not change during weight loss despite the decrease in MER. A second issue is the fact that for loss of lean tissue mass to be minimised during weight loss, intake of dietary protein must be adequate [14]. However, it is not known which essential amino acids are most limiting in a purpose-formulated weight management diet. Therefore, additional studies are now required to assess adequate intake of essential nutrients during weight loss.

The majority of essential nutrients were fed at intakes (per kg BW^0.75^/day) above the guidelines recommended by NRC, throughout the weight loss period. However, there were some important exceptions to this, most notably selenium, choline, methionine and cysteine. At first glance, results were most concerning for selenium, since daily intakes for this mineral were less than AI in all cases. Selenium is involved in antioxidant pathways as well as both thyroid and immune system function, with deficiency reportedly causing anorexia, depression, dyspnoea, and coma [24]. None of these signs were evident in any of the dogs of the current study. Excessive dietary selenium intake can also have adverse effects in dogs [24]; further, both AAFCO and FEDIAF have set legal limits for selenium supplementation. Thus, it can be immensely challenging to formulate a diet appropriately for selenium content. A second challenge with selenium is the fact that optimal intake is not as easily determined as for other nutrients. Most notably, NRC does not report MR for selenium in dogs and, to the authors’ knowledge, no studies have examined this, partly because it can be difficult to measure selenium status accurately in animals. Nonetheless, a recent *in vivo* study in obese dogs, that used the same diet as for the current work, did not reveal any decrease in selenium status during weight loss. In fact, urinary selenium excretion was greater after weight loss, compared with before, perhaps suggesting that requirements for this essential nutrient might actually decline during weight loss [18]. Therefore, although less than NRC AI, the intake of selenium in all dogs was probably sufficient.

Choline is a vitamin-like substance that is reportedly involved in neurotransmission, hepatic lipid metabolism, coagulation, as well as acting as a methyl donor [15]. In dogs, deficiency of choline causes hypocholesterolaemia, vomiting, fatty liver disease, and death [25]. A previous theoretical study suggested that intake of choline might be at risk of deficiency if marked caloric restriction was required during weight loss [19]. In the current study, the daily choline intake of most dogs (24/27, 89 %) was less than the NRC 2006 RA for some of their period of weight loss, although intake was less than the suggested AI in only two dogs (7 %). However, requirements for choline have not been well established in dogs, and the current AI for choline is based on data from studies conducted over 50 years ago [16]. Therefore, it is unclear as to whether daily choline intake during the current study was actually deficient. That said, a recent study, that used the same diet as for the current work, demonstrated a 16 % decrease in plasma choline concentrations during weight loss in obese dogs [18]. Given that choline is a relatively easy nutrient to supplement and there are minimal toxicity concerns, increasing the choline content of canine weight management diets would seem be sensible until more data on choline requirements are available.

Similar to the issues for choline, the intake of methionine and cysteine were less than RA in 12 (44 %) and less than MR in 2 (7 %) of the dogs undergoing weight loss in the current study. As with choline, these amino acids were also identified as ‘at risk’ in a recent theoretical study [19]. Methionine is a sulphur-containing amino acid that is not only required for protein synthesis, but also forms part of the coenzyme s-adenosyl methionine [26]. As a result, its deficiency can result in various metabolic aberrations [26]. Cysteine is a critical amino acid for maintaining the secondary structure of compounds such as glutathione and those required for hair synthesis. Cysteine is synthesised from methionine and, therefore, both amino acids are typically considered together when determining requirements. When methionine is deficient in the diet, there is an immediate decrease in food intake, and severe weight loss [27]. Whilst all dogs lost weight during the study, this was not likely to be due to methionine or cysteine deficiency because food intake was never affected and weight loss occurred in a controlled fashion and was never excessive (i.e. < 3 % per week). Puppies fed a methionine-deficient diet also develop dermatological lesions such as erythema, footpad necrosis and hyperkeratosis [28], while adult dogs fed a diet with borderline methionine and taurine content, develop gallstones [29, 30]. None of the dogs in the current study, or indeed from the larger cohort of dogs seen at our weight management clinic, developed any dermatological signs at any stage during or after their period of weight loss. Given that abdominal ultrasonography was not performed, it is unclear as to whether or not gallstones had developed. That said, this possibility is less likely, given that the diet was also supplemented with taurine (which can partially substitute for methionine and cysteine), and none of the dogs were taurine deficient (data not shown). Further, no signs were noted pertaining to liver disease, and clinicopathological markers of biliary system disturbance (e.g. bilirubin, and liver enzyme activity) were unchanged after weight loss. Finally, given the requirement of both methionine and cysteine for protein synthesis, the effect of daily intake on change in lean tissue mass and also serum albumin concentration were assessed, and no associations were seen with either. Nonetheless, as with choline, supplementing methionine and cysteine in the current diet by 15 % would ensure that daily intake is greater than NRC MR. Such a reformulation would be sensible until further data are available clarifying the safety of intakes of the level in the current study.

There were a number of other nutrients where intake was less than the RA suggested by the NRC [15]. These included tryptophan, magnesium, and potassium. The significance of these observations is not known since intake was always above MR throughout weight loss, there were no signs of deficiency for any of these nutrients, and no association was seen with changes in lean tissue or serum albumin.

As with all studies, a number of limitations exist. Firstly, the data were collected retrospectively, and dogs included were drawn from a larger population. Given the eligibility criteria used, and most notably the duration of the weight loss period, a number of dogs were excluded since they reached target weight within 6 months. Other dogs were excluded because different weight loss diets had been used, clinical data were missing, the dogs either had another disease, or they were on concurrent drug therapy. It is possible that, by excluding dogs in this way, the results might have been unfairly biased. That said, dogs were not excluded for becoming sick or ill, perhaps the most important outcome factor of interest for the study. Further, to the authors’ knowledge, no nutrient deficiencies have ever been recognised in any dog that has attended the weight management clinic where the study was conducted, and this includes the dogs from the current study. Nonetheless, it would be worth considering a prospective study, to confirm the current findings in a cohort of obese dogs undergoing weight loss.

Given that client-owned dogs were used, a second limitation was the diversity in the population of dogs used, which is arguably more marked than would have been the case for a study undertaken on dogs from a research colony. Not only was the population more diverse, but also the environment in which the dogs were kept was more variable, not least with regard to controlling food intake. To offset this, owners measured food out precisely using kitchen scales, maintained a diary record, and dogs were only included dogs if there was no evidence of poor compliance i.e. recorded feeding of additional foodstuffs. That said, under-reporting is a concern with the use of food questionnaires [11, 31] and, as a result, it is possible that some dogs received additional food, causing errors in estimation of adequate intake. Whilst this is a notable limitation, the use of client-owned dogs means that the results are arguably more representative for the target population of interest, than using dogs from a research colony.

A third limitation regarding the population was the fact that it was very variable in terms of breed, age gender, presence of concurrent diseases, and in outcome. For this reason we applied very strict eligibility criteria, for instance measurement of body composition by DEXA. As a result, the final population was closely monitored, well phenotyped, and all data were complete.

A fourth limitation was the fact that, since the study was conducted over a number of years and there was no actual analysis of the batches of food used. Instead, the nutrient intake for each dog was based upon the average nutrient content of the diet, based upon analysis. Thus, actual nutrient intake might have differed from that reported in the current study. Further, whilst we observed no obvious clinical signs of malnutrition, based upon physical examination, the outcomes measured might not have adequately assessed nutrient status or detected subclinical deficiencies. For instance, plasma nutrient status was not measured. However, such a study has recently been performed, in a similar cohort of dogs [18], which demonstrated no change in the plasma concentrations of most key nutrients during weight loss.

A final limitation relates to generalisability of the data to canine weight loss programmes in general. Given the use of client-owned dogs, results should be broadly representative of weight loss programmes using this or similar diets in practice although, since the final population was small, subtle individual issues with a particular breed or dog type might have been missed. Further, the population came from a referral clinic and so might not be typical of the usual dogs undergoing weight management. Moreover, only a single weight loss diet was used and, thus, findings might not be generalisable to other weight loss diets, especially those from other manufacturers. Therefore, it would be worth considering individual validation of other weight loss strategies in the future.

## Conclusion

In conclusion, the current study reports daily intakes of essential nutrients in a cohort of obese dogs that successfully completed a weight loss regime of at least 6 months’ duration, and showed no signs of nutrient deficiency. For some essential nutrients, such as selenium, choline, methionine and cysteine, daily intake was borderline in some dogs during weight loss.

## Additional files

Additional file 1:
**STROBE 2007 (v4 Statement—Checklist of items that should be included in reports of cohort studies.** (PDF 89 kb) Additional file 2:
**Study data.** (XLSX 254 kb)

## Abbreviations

AAFCO: Association of american feed control officials
AI: Adequate intake
BCS: Body condition score
BW: Body weight
DEXA: Dual-energy X-ray absorptiometry
DM: Dry matter
FEDIAF: European pet food industry federation
HCT: Haematocrit
LC-MS/MS: Liquid chromatography-tandem mass spectrometry
MR: Minimum requirement
NRC: National research council
RA: Recommended allowance
